# Differential Virulence of West Nile Strains for American Crows

**DOI:** 10.3201/eid1012.040486

**Published:** 2004-12

**Authors:** Aaron C. Brault, Stanley A. Langevin, Richard A. Bowen, Nicholas A. Panella, Brad J. Biggerstaff, Barry R. Miller, Nicholas Komar

**Affiliations:** * Centers for Disease Control and Prevention, Fort Collins, Colorado, USA:; † University of California, Davis, California, USA;; ‡ Colorado State University, Fort Collins, Colorado, USA

**Keywords:** West Nile virus, American Crows, strains, mortality, virulence, Kunjin, research

## Abstract

Crow deaths were observed after West Nile virus (WNV) was introduced into North America, and this phenomenon has subsequently been used to monitor the spread of the virus. To investigate potential differences in the crow virulence of different WNV strains, American Crows were inoculated with Old World strains of WNV from Kenya and Australia (Kunjin) and a North American (NY99) WNV genotype. Infection of crows with NY99 genotype resulted in high serum viremia levels and death; the Kenyan and Kunjin genotypes elicited low viremia levels and minimal deaths but resulted in the generation of neutralizing antibodies capable of providing 100% protection from infection with the NY99 strain. These results suggest that genetic alterations in NY99 WNV are responsible for the crow-virulent phenotype and that increased replication of this strain in crows could spread WNV in North America.

## Abstract 2

Increased viremia and deaths in American Crows inoculated with a North American West Nile viral genotype indicate that viral genetic determinants enhance avian pathogenicity and increase transmission potential of WNV.

West Nile virus (WNV, *Flaviviridae*: *Flavivirus*) is maintained in nature by transmission between mosquitoes and birds and has an extensive geographic range, including Europe, Africa, the Middle East, southern Asia, and Australia (*1*). In 1999, WNV was identified in North America (*2*) and has become the leading cause of arboviral encephalitis in humans and horses (*3*), as well as having been implicated in deaths of members of at least 198 bird species (*4*). Corvids, including the American Crow (*Corvus brachyrhynchos*), appear to be most susceptible (*5**,**6*), and corvid deaths have subsequently been used as a sentinel to track the spread of the virus (*7*).

Experimental injection of American Crows with the North American genotype of WNV (NY99 strain) has confirmed its highly pathogenic phenotype. Mean peak viremia titers in American Crows exceed 9 log_10_ PFU/mL in sera, with 100% deaths within 6 days postinfection (dpi) (*5*). With the exception of bird deaths in Israel (*8*), where a strain 99.8% similar to the NY99 genotype has circulated since 1997 (*9*), no bird deaths have been reported during numerous well-characterized WNV epidemics in North Africa (*10*), Europe (*11**–**13*), Russia (*14*), and South Africa (*15*). A closely related virus that circulates in Australia (Kunjin [KUN]) has never been associated with outbreaks of human or animal diseases, including bird diseases, nor have bird deaths been reported from enzootic transmission foci in Africa, where a virus that shares 96.5% nucleotide identity with the NY99 strain has previously been isolated (*16**,**17*). Possible explanations for the lack of reporting of bird deaths before 1998 include the following: failure to identify bird deaths in other regions, a higher susceptibility to WNV-induced disease among North American birds, or the fact that the North American WNV strain possesses increased avian virulence determinants. Additionally, the possible immunologic cross-protection of birds with lesser virulent strains could be a factor that has limited the identification of bird deaths outside the Middle East. Immunologically naïve bird populations in North America could be at an increased risk of acquiring severe disease.

The close genetic relatedness of the North American WNV genotype with the bird-pathogenic Israeli WNV strain suggests differential avian pathogenicity among WNV strains (*9*). To evaluate whether WNV-associated deaths in American Crows was due to infection by a more virulent genotype, we injected American Crows with NY99, a closely related WNV strain from Kenya (KEN) and a more distantly related WNV strain from Australia (KUN) and monitored viremia titers and illness. In addition, birds that survived challenge with the KEN or KUN viruses were challenged with a lethal dose of the NY99 strain to assess development of a cross-protective immunologic response.

## Materials and Methods

### Viral Strains and Birds Used

The lowest passage WNV available were used for crow virulence studies to avoid incorporating confounding cell-culture–related genetic substitutions. The NY99 isolate used was originally isolated from an American Crow brain (strain NY99-4132) and was subsequently passaged once in Vero cells before being used for these studies. The Kenya-3829 (KEN) isolate was made from a pool of male *Culex univittatus* mosquitoes (*16*) and passaged twice in Vero cells. The Kunjin (KUN-6453) isolate was made from *Cx. annulirostris* mosquitoes and was passaged once in Vero cells and once in hamster kidney cells (Table 1). After-hatch-year American Crows were obtained by using net traps with the assistance of the Kansas Department of Wildlife Resources. The crows were banded and transported to Fort Collins, Colorado, where they were housed in the Colorado State University Animal Disease Laboratory in groups of two in 1-m^3^ cages. Crows were fed a combination of ground corn and dried cat food and dog food.

**Table 1:** West Nile viral strains used for virulence studies in American Crows

Virus	Strain	Source	Passage history^a^	Location	Genetic lineage^b^
NY99	NY99-4132	American Crow (brain)	V1	USA	I
KEN	KEN-3829	*Culex univittatus*	V2	Kenya	I
Kunjin (KUN)	KUN-6453	*Cx*. *annulirostris*	V1, BHK1	Australia	I

^a^Viruses were propagated in Vero (V) or baby hamster kidney (BHK) cells. Numbers following passage source represent the number of viral passages. ^b^Genetic lineages as reported previously (*9*).

### Detection of Preexisting Flaviviral Antibodies

To confirm that crows had not previously been exposed to WNV or another endemic flavivirus, St. Louis encephalitis virus (SLEV), crows were bled before injection and serum-tested by plaque reduction neutralization assays (PRNTs) with WNV and SLEV viruses. Serum was diluted 1:5, heat inactivated at 56°C for 30 min, and incubated with an equal volume of virus (SLEV; strain TBH-28) and WNV (strain NY99-4132) to a final concentration of 100 PFU/0.1 mL. Samples were incubated at 37°C for 1 h, and 0.1 mL of each was added to a confluent monolayer of Vero cells in 6-well plates (Costar Inc., Cambridge, MA). After incubation for 1 h, cell monolayers were overlaid with 0.5% agarose; a second overlay containing 0.005% neutral red was added 48 h later. Plates were read 1–2 days after addition of the second overlay. A 90% reduction in PFU, as compared to the serum-negative control, was used as the determinant of neutralization. Detection of any neutralizing activity to either SLEV or WNV within the serum of any crow precluded use for experimental inoculation.

### Virus Injection

Viral stocks were diluted to 3.2 log_10_ PFU/0.1 mL in minimal essential media (MEM) containing no fetal bovine sera (FBS). One hundred microliters of the diluted stocks was subcutaneously injected on the breast region of eight American Crows in four infection groups. Crows were injected with 1) NY99, 2) KEN, 3) KUN WNVs, or 4) with a media-only injection that served as a virus-negative control. In addition, a higher dose inoculum of 3.8 log_10_ PFU/0.1 mL was prepared for injection of a fifth group of crows with KEN WNV. All crows were examined for signs of disease twice daily for 14 days after injection and bled once daily from 1 to 7 dpi for characterization of viremia. Blood samples were collected from the jugular or brachial vein by using a 26-gauge needle; 0.2 mL of blood was added to 0.9 mL of MEM supplemented with 20% FBS to obtain approximately a 10^–1^ serum dilution. Coagulation was allowed to take place at room temperature for 30 min, at which point samples were placed on ice and spun at 3,700 x *g* for 10 min to pellet clotted cells. The supernatants from these samples were frozen at –80°C until samples were titrated for infectious units.

### Assaying for Infectious Virus

Infectious virus was assayed by plaque formation on monolayers of Vero cells. Briefly, serial 10-fold dilutions of serum were added to Vero cells that were overlaid as described previously for PRNTs. PFU were enumerated at 3 dpi and multiplied by the dilution factor to determine viral titers per mL serum. Initial 1:10 dilution of serum as well as the use of 200 µL of the lowest dilution, resulted in a limit of viral PFU detection of 1.7 log_10_ PFU/mL serum. Inocula for all three viruses were back-titrated by plaque assay in order to confirm the uniformity of the doses administered.

### Determination of Cross-Protection

Blood (0.6 mL) was drawn at 14 dpi to determine the levels of WNV-specific antibodies and cross-neutralization by using a 2-way β PRNT with homologous and heterologous WNV strains. Briefly, twofold dilutions of bird serum samples were incubated at 56°C for 30 min and mixed with either NY99, KEN, or KUN viruses. Samples were allowed to incubate for 1 h at 37°C, at which point the samples were injected onto Vero cells and overlaid as previously described for PRNT. Plaques were counted, and neutralization was reported as a 90% reduction in plaque formation as compared to the results for the serum-negative control.

Crows that survived through 14 dpi were subsequently challenged with 3.2 log_10_ PFU of NY99 virus from the same seed that was used for the initial infection of the experimental NY99 infection group. Crows were bled daily through 7 dpi and were held through 11 dpi, at which point an additional 0.6 mL of blood was drawn to assess modulations in neutralizing activity after secondary challenge. Serum samples from the seven daily bleedings were diluted 1:10 in MEM diluent, spun, immediately assayed for the presence of infectious virus on Vero cells, and then stored at –80°C. Samples demonstrating virus were thawed and titrated on Vero cells as described above. Additionally, serum drawn at the end of the time course was assayed for antibody by PRNT.

### Statistical Analyses

Statistical analyses were performed on peak viremia level, duration of viremia, day of viremia onset, and day of death. All analyses with the exception of day of death were performed by analyses of variance (ANOVA). Multiple comparisons, i.e., confidence intervals (CI) for the difference of means, were performed by using Tukey's highest significant difference (HSD) adjustment for comparisons of means. Because only two virus groups had birds that died, the day-of-death comparisons were analyzed by using a Student *t* test with Welch's modification for unequal variances. Proportions of illness and death were compared with the Fisher exact test.

## Results

**Figure 1:**
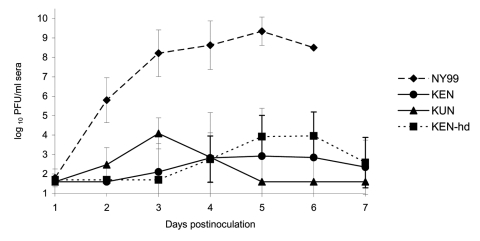
Viremia profiles for West Nile virus (WNV)–infected American Crows after injection of 1,500 PFU of KUN or KEN/NY99 WNV. Viral titers were determined by plaque formation on Vero cells and represented as geometric means. A detection limit of >1.7 log_10_ PFU/mL crow serum was determined. Bars represent standard deviations (SD) of the mean. hd, high dose.

Flaviviral antibodies were not detected in any of the preinoculation serum samples assayed by PRNT. Therefore, all captured American Crows were used for experimental inoculation. Peak viremia titers ranging from 6.7 log_10_ to 10.7 log_10_ PFU/mL serum (mean peak viremia titers = 9.2 log_10_ PFU/mL serum, 95% CI 8.2 log_10_ PFU/mL serum–10.2 log_10_ PFU/mL serum) developed in all crows injected with the NY99 WNV genotype (Figure 1). Onset of viremia occurred within 24 h for three of the eight crows injected with NY99 and was present in all eight birds within 48 h postinjection (mean onset of viremia = 1.8 dpi, 95% CI 1.4 dpi–2.1 dpi) (Table 2). Mean onset of viremia and mean peak viremia titers differed significantly among the virus groups (mean onset, F = 31.6, df = 3,22, p < 0.001; mean peak viremia, F = 74.9, df = 2,21, p < 0.001). In contrast to the NY99-infected crows, detectable viremia (>1.7 log_10_ PFU/mL sera) developed in two crows infected with the KEN WNV. The onset of viremia in these two birds was delayed until 3 dpi and 4 dpi, and the mean peak viremia level was lower than that of the NY99 infection group (7.5 log_10_ PFU/mL) (difference of mean onset of viremia = 1.8 dpi, 95% CI 0.4 – 3.1). When the inoculum dose was increased to 3.8 log_10_ PFU for the KEN strain, viremia developed in all eight of the crows, with peak titers ranging from 4.2 to 6.1 log_10_ PFU/mL serum (mean = 4.9 log_10_ PFU/mL serum, 95% CI 4.3–5.4 log_10_ PFU/mL serum). The onset of viremia was delayed in the higher dose KEN group compared to the NY99 infection group (mean = 4.5 dpi, 95% CI 3.9–5.1 dpi; difference of mean onset of viremia = 2.8 dpi, 95% CI 1.9–3.6 dpi). In all eight crows inoculated with 3.2 log_10_ PFU of KUN virus, peak viremia titers were 2.7–4.9 log_10_ PFU/mL serum (mean = 4.2 log_10_ PFU/mL, 95% CI 3.5–4.8 log_10_ PFU/mL serum). Onset of viremia relative to the NY99-infected crows was slightly delayed, with a mean onset at 2.4 dpi (95% CI 1.9–2.8 dpi) (difference of mean onset of viremia = 0.6 dpi, 95% CI 0.2–1.5 dpi) (Figure 1). Viremia developed in KUN-infected crows, lasting from 1 to 5 days with a mean duration of 3 days (95% CI 1.9–4.1 days). This finding differs qualitatively from the NY99- and KEN-infected birds, which sustained viremia for at least 4 days; viremia levels ceased only when the bleeding time course was halted or at time of death; the differences between the viremia durations for the KUN-infected crows and the NY99 and KEN groups were not statistically significant when adjustments were made for multiple comparisons.

**Table 2:** Clinical profile of American Crows infected with WNV strains NY99 (strain NY99-4132), KEN (strain KEN-3829), and KUN (strain KUN-6453)^a^

Virus group	Mortality: no. died/N (%)	Morbidity: no. ill/N (%)	Mean day of death ± SD	Mean peak viremia^a^ ± SD (mean duration ± SD) (n)	Mean day of peak viremia^a^ ± SD
NY99	8/8 (100)	8/8 (100)	5.1 ± 0.6	9.2 ± 1.2 (4.2 ± 0.7) (8)	4.3 ± 0.9
KEN	1/8 (12.5)	2/8 (25)	9 ± NA	7.5 ± 0 (4.5 ± 0.7) (2)	5.0 ± 1.4
KEN-hd^b^	2/8 (25)	3/8 (38)	10.5 ± 2.1	4.8 ± 0.6 (3.1 ± 0.8) (8)	5.5 ± 0.9
KUN	0/8	0/8	NA	4.2 ± 0.8 (1.8 ± 0.5) (8)	3.3 ± 0.7
Control	0/8	0/8	NA	NA	NA

^a^Viral titers were expressed as the log_10_ PFU/mL of crow sera as determined by plaque assay on Vero cells. ^b^hd, high-dose group (6,000 [3.8 log_10_] PFU); NA, not applicable.

**Figure 2:**
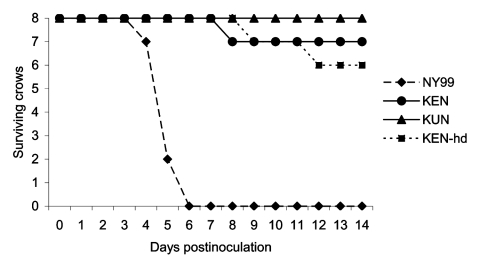
Survivorship of eight American Crows, each injected with 3.2 log_10_ PFU of NY99, KEN, or KUN virus. An additional eight crows were injected with a high dose (hd) of the KEN virus (3.8 log_10_ PFU). Crows were monitored daily for signs of disease through 14 dpi. No deaths were found within the control group (data not shown).

All crows in the NY99 group died by dpi 6 (Figure 2). Signs of illness (unresponsiveness, anorexia, weight loss), signs of encephalitis (shaking, convulsion, ataxia), or both developed in all NY99-infected crows. In addition, hemorrhage from oral and cloacal cavities was evident in five (62.5%) of the eight crows in the NY99 group. One crow died of infection with NY99 at 4 dpi, five at 5 dpi, and the remaining two at 6 dpi (Figure 1). Only one crow (12.5%) died of infection with the KEN virus with the 3.2 log_10_ PFU injection. When the dose was increased to 3.8 log_10_ PFU, 2 (25%) crows did not survive the infection. Regardless of the dose administered, the crows infected with the KEN virus demonstrated a reduced mortality rate (p < 0.001), compared to that of the NY99 virus. Virus was isolated from the brains of the small subset of crows that died of infection with the KEN strain (data not shown). In addition to the three deaths from the KEN WNV genotype, an additional two crows showed signs of illness, yet survived through 14 dpi (Table 2). No illness or death was identified within the KUN infection group, yielding a significant difference from the NY99 infection group (p < 0.001), but the KUN group was not statistically differentiated from the KEN WNV infection groups (p = 0.53).

**Figure 3:**
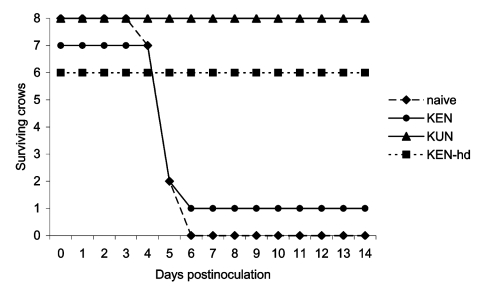
Survivorship of American Crows previously immunized with West Nile virus (WNV)-KUN or WNV-KEN viruses after injection with 1,500 PFU of NY99 WNV. hd, high dose.

**Figure 4:**
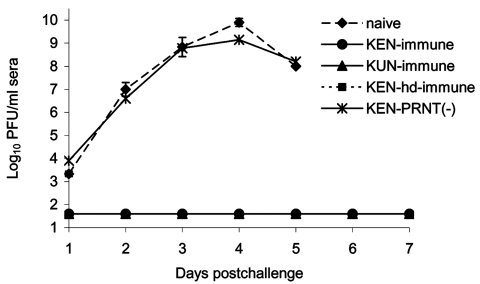
Viremia production of American Crows previously immunized with West Nile virus (WNV)-KUN or WNV-KEN viruses after injection with 3.2 log_10_ PFU of NY99 WNV. No detectable levels of viremia (>1.7 log_10_ PFU/mL crow serum) developed in the KUN virus–immunized crows (0/8). Data points for the naïve (unexposed to WNV) crows challenged with the NY99 virus represent the mean of three samples chosen randomly. Bars represent standard deviations (SD) of the mean. hd, high dose; PRNT, plaque reduction neutralization assay.

None of the eight crows previously challenged with KUN virus had detectable illness after secondary challenge with 3.2 log_10_ PFU of NY99 virus (Figure 3), which clearly indicates a cross-protective immune response against NY99; the lower 95% confidence limit on cross-protection probability was 0.63. In fact, viremia was not detected in any of the eight crows rechallenged with the NY99 WNV on any of the 7 dpi (Figure 4). PRNTs demonstrated a homologous neutralization response in all eight of the crows for KUN virus (Table 3). Heterologous titers against NY99 virus were equal to or only twofold lower than those against KUN virus.

**Table 3:** Cross-neutralization immune response of American Crows at 14 days postinfection with either KEN or KUN viruses

Sample no.	Inoculation	NY99	KEN	KUN	Difference
Crow 8	KEN	640^a^	**640** ^b^	NT	0
Crow 1	KUN	80	NT^c^	**160**	2-fold
Crow 2	KUN	80	NT	**80**	0
Crow 3	KUN	160	NT	**320**	2-fold
Crow 4	KUN	40	NT	**80**	2-fold
Crow 5	KUN	40	NT	**40**	0
Crow 6	KUN	20	NT	**20**	0
Crow 7	KUN	80	NT	**80**	0
Crow 8	KUN	80	NT	**160**	2-fold

^a^Values represent the greatest reciprocal dilution in which >90% plaque inhibition was achieved as compared to sera-negative control cultures. ^b^Homologous titers are depicted in **bold** print. ^c^NT not tested; KEN, West Nile virus strain from Kenya; KUN (Kunjin), West Nile virus strain from Australia.

Only one of the seven crows from the lower dose (3.2 log_10_ PFU) KEN WNV inoculation group survived rechallenge with the NY99 strain (Figure 3). Sera drawn before the NY99 rechallenge from all crows within this group demonstrated that an immune response had developed in one crow (the single survivor). This crow demonstrated illness after the original KEN WNV challenge and was one of the two crows that had detectable viremia levels and subsequently exhibited a homologous protective antibody titer that was indistinguishable from its heterologous titer against the NY99 virus (1:640) (Table 4). The six KEN-infected survivors that did not become viremic from the original KEN viral challenge were devoid of detectable neutralizing antibody titers and had unmodified infections after the NY99 challenge. The viremia profile and clinical outcome (Figure 3 and Figure 4) were indistinguishable from infection of naïve birds: five crows died on 5 dpi and an additional crow died on 6 dpi. The single surviving crow that had demonstrated a 1:640 heterologous PRNT titer against NY99 WNV did not manifest a NY99 viremia level and had an unmodified 1:640 PRNT titer after the NY99 challenge. All crows from the group that received the higher dose of KEN generated KEN viremia titers and exhibited homologous PRNT titers (1:1,280–2,560) that were indistinguishable (less than fourfold difference) from heterologous titers against the NY99 virus. Neither clinical disease nor NY99 viremia levels were identified in these crows after secondary challenge with the NY99 virus, but neutralizing antibody titers increased up to 16-fold. The rise in PRNT titer was most likely the result of secondary infection or exposure; however, no control American Crows (to which a secondary challenge was not administered) were assayed for elevated PRNT titers at 24 dpi.

**Table 4:** Cross-neutralization immune response of American Crows 24 days postinfection (dpi) with either KEN or KUN viruses^a^

Sample no.	Inoculation	NY99	KEN	KUN	Difference
Crow 8	KEN	640^b^	**640** ^c^	NT	0
Crow 1	KUN	160	NT^d^	**320**	2-fold
Crow 2	KUN	320	NT	**320**	0
Crow 3	KUN	160	NT	**160**	0
Crow-4	KUN	160	NT	**320**	2-fold
Crow 5	KUN	160	NT	**160**	0
Crow 6	KUN	320	NT	**320**	0
Crow-7	KUN	640	NT	**640**	0
Crow 8	KUN	640	NT	**640**	0

^a^Following secondary NY99 challenge at 14 dpi. ^b^Values represent the greatest reciprocal dilution in which >90% plaque inhibition was achieved as compared to sera-negative control cultures. ^c^Homologous titers are depicted in **bold** print. ^d^NT not tested; KEN, West Nile virus strain from Kenya; KUN (Kunjin), West Nile virus strain from Australia.

Sequence analyses of the coding differences between the NY99 and KEN viruses (Table 5) were performed on a NY99 virus (that had undergone an additional 2 Vero cell passages) to assess the possibility that limited cell-culture propagation could have resulted in attenuating genetic substitutions found between the KEN and NY99 genotype. These analyses did not demonstrate any genetic modification at any of the KEN or NY99 variable sites, further indicating that the genotype is stable for up to at least 3 passages and that the attenuated phenotype of the KEN or KUN viruses was unlikely to be the result of an additional tissue culture passage.

**Table 5:** Amino acid differences between the NY99 and KEN West Nile virus strains^a,b^

Viral gene	Amino acid position	NY99	KEN
**Capsid** ^a^	**3**	**Leu**	**Asn**
**Capsid**	**8**	**Val**	**Ala**
**Envelope**	**126**	**Ile**	**Thr**
**Envelope**	**159**	**Val**	**Ile**
NS1	70	Ala	Ser
NS2a	52	Thr	Ala
NS2b	103	Val	Ala
NS3	249	Pro	Thr
NS3	356	Thr	Ile
NS4a	85	Ala	Val
NS4b	249	Glu	Asp

^a^Source (*17*). ^b^KEN, West Nile virus strain from Kenya; Leu, leucine; Val, valine; Ile, isoleucine; Ala, alanine; Thr, threonine; Pro, proline; Glu, glutamine; Asn, asparagine; Ser, serine; Asp, aspartic acid. ^c^Variable structural amino acid residues have been designated by **bold** text.

## Discussion

Viremia levels observed in these studies confirm previous observations that American Crows have the potential to serve as amplification hosts for the NY99 genotype of WNV but suggest that corvids may not be important hosts for alternative WNV genotypes because of substantially reduced viremia titers that would not favor efficient virus transmission. Furthermore, these results demonstrate that viral-encoded determinants of avian pathology that are absent from KEN and KUN viruses exist in the NY99 virus. The viremia levels observed in crows inoculated with the KEN or KUN viruses were significantly lower than and delayed in their onset compared to those seen after inoculation with the NY99 strain. These data demonstrate that the differential pathogenic phenotypes of the WNV strains are the result of viral genetic differences that encode particular virulence determinants. Despite the finding that mouse virulence of the NY99 and KUN WNV strains (*18*) correlates well with the virulence phenotype identified in crow experiments here, experimental infection of mice with the KEN WNV strain did not demonstrate an attenuated phenotype (D.W.C. Beasley and A.D.T. Barrett, pers. comm.). This observation indicates that differential pathogenic mechanisms could modulate virulence in disparate vertebrate hosts.

Elevated viremia level could be a predominant factor for severe clinical outcome. KUN and KEN WNV-infected crows in which clinical signs did not develop did not manifest peak viremia titers >6 log_10_ PFU/mL; however, peripheral titers exceeded this level for the three crows in which neurologic symptoms and death occurred. Additionally, viremia levels of all crows injected with NY99 surpassed this level, which suggests that once a peripheral circulatory threshold titer is achieved, virus is capable of accessing the nervous system through a nonspecific mechanism. Intracerebral injection of mice with WNV strains differing in neuroinvasive capacity has demonstrated uniform lethality, indicating that the ability to enter the nervous system and not neurovirulence, is instrumental for virulence of WNV strains (*18**,**19*). If this phenomenon is true for WNV strains in crows, then the mechanism by which the crow-virulent genotype achieves extremely high peripheral titers must be elucidated. Viruses capable of replicating to higher titers could result from a unique access to cell types that facilitate high-titer replication through more efficient receptor-envelope interactions, viral replicase differences that increase replication efficiency within host cells, decreased sensitivity to host innate immunologic responses, or by altering the physiological host responses such as fever.

Immunologic status of a host can play an important role in limiting disease expression. WNV that are capable of inducing substantial levels of viremia and neuroinvasion of immunodeficient mice do not necessarily cause viremia or enter the neural tissues of mice with competent immune systems (*19*). Studies have demonstrated that previous infection with heterologous flaviviruses reduces the incidence of encephalitis and can provide protection from fatal WNV challenge in a hamster model for WNV pathogenesis (*20**,**21*). In contrast, a neutralization study performed with WNV strains of different lineages demonstrated that neutralizing antibodies against an Indian WNV strain provided poor protection against a South African WNV strain (*22*). Our results demonstrated that prior immunization with KUN virus can provide protection from lethal NY99 challenge in crows. Crows in which a detectable level of viremia did not develop from the initial KEN viral challenge exhibited viremia levels and death rates indistinguishable from NY99-infected naïve crows. Crows injected with the higher doses, which led to productive infections with the KEN virus, produced neutralizing-antibody titers that were protective against lethal NY99 challenge. The cross-neutralization of WNV strains suggests that areas in which WNV virus is endemic could be much less susceptible to invasion by the crow-virulent NY99 genotype.

The effect that endemic flaviviruses such as SLEV has on the genetic stability of WNV in North America remains unclear; however, the fact that WNV and SLEV are distinguishable serologically through PRNT (*23*) and that WNV activity within the United States has occurred sympatrically within SLEV transmission foci (*3*) suggest that SLEV seroprevalence in birds has little impact on WNV transmission. Previous studies have demonstrated in a flaviviral pathogenesis hamster model that previous exposure to SLEV can significantly reduce WNV viral titers (*21*). Future experiments are warranted to determine if such protection is afforded in avian species.

Experimental inoculation with an Egyptian WNV strain has demonstrated deaths in sparrows and crows (*24*), providing evidence that bird deaths could result from natural infection with alternative WNV genotypes. Despite this fact, no bird deaths were reported during a well-described Egyptian epidemic involving the same viral strain used to experimentally inoculate these birds (*10*). Our results demonstrated that low numbers of deaths can occur from infection with alternative WNV strains, but the NY99 WNV genotype is significantly more virulent for American Crows. This result, coupled with the finding that similar pathogenicity was identified between the NY99 and KEN WNV in house sparrows (*25*), indicates the dual role of viral pathogenic phenotype and host susceptibility for the expression of virulence in a particular bird species. Differential susceptibility of mouse strains for WNV infection has been identified and correlated with immunologic gene expression (*26*). Future experimental inoculation of Old World corvids with differential WNV genotypes would be useful to assess the role that host susceptibility has on the emergence of WNV genotypes in different geographic regions.

The mutations that encode the determinants for differential crow virulence are currently unknown. In crows inoculated with a recombinant virus containing WNV structural genes and nonstructural (NS) genes of yellow fever virus (YFV), viremia did not develop (*27*). The fact that the parental YFV-17D vaccine strain did not replicate to detectable levels in chickens (*28*) indicates that flaviviral NS gene regions could modulate viral replication in birds. Analysis of the complete genomes of the NY99 and KEN WNV has identified a maximum of 11 amino acids (Table 5) and 22 nucleotides from the 3´NCR that could mediate this phenotype (*17*). Seven (64%) of the 11 amino acid differences between these viruses resided with the NS gene region. The close genetic identity between the KEN and NY99 WNV genotypes makes this an optimal system for the systematic identification of genetic elements that encode viral pathogenic determinants. Studies are under way to identify the specific viral genetic determinants of crow virulence through the use of infectious cDNAs generated from both the NY99 and KEN WNV genotypes.
